# Some Points in the Treatment of Diphtheritic Laryngeal Obstruction.—II

**Published:** 1911-07-22

**Authors:** A. Knyvett Gordon

**Affiliations:** formerly Medical Superintendent of Monsall Hospital and Lecturer on Infectious Diseases in the University of Manchester.


### Hospital Clinics.

### SOME POINTS IN THE TREATMENT OF DIPHTHERITIC LARYNGEAL OBSTRUCTION.—II.

By A. KNYVETT GORDON, M.B. Cantab., formerly Medical Superintendent of Monsall Hospital and Lecturer on Infectious Diseases in the University of Manchester.

When we have decided to operate we have the choice of two procedures: tracheotomy and intubation of the larynx.

### The Operation of Tracheotomy.

For tracheotomy the child is wrapped in a blanket which encircles the trunk and arms just below the shoulders and extends below the feet; this is secured by three large safety-pins. A sand bag or other hard substance is placed underneath the shoulders, and the head is extended over the end of a convenient table. One assistant holds the head, taking special care not to allow it to roll from side to side, and another secures the body and legs. A general anæsthetic is, above all things, to be avoided, and is moreover unnecessary, as slightly asphyxiated children do not feel the pain of an incision but are terrorised by the suffocating sensation of chloroform. In older children, cocaine and adrenalin may be injected over the site of the incision, but this is not often necessary. The operator now feels for, and places his left forefinger on, the lower edge of the thyroid cartilage, and makes an incision downwards for an inch, or an inch and a half, in the middle line of the neck. The cricoid cartilage in most young children cannot be felt through the skin, but the thyroid edge is always well marked. The left forefinger is now thrust into the incision and the cricoid cartilage defined. This is always possible, and the secret of success now lies in keeping the finger on the cricoid while the deep incision is being made. This should be an upward cut with the knife held vertically, and should go right through the fascia and open the trachea as fully as possible—that is to say, up to the cricoid cartilage, and in very young children with fat necks it is usually best to include this in the tracheal cut.

### Hæmorrhage.

Free hæmorrhage now generally ensues, but no attempt should be made to arrest it with pressure forceps, but rather by the more satisfactory method of dilating the tracheal incision so that free respiration may be established. Inasmuch as the coughing reflex has not been diminished by a general anæsthetic, any blood which might otherwise find its way into the trachea is coughed out. With the left forefinger still on the cricoid, a pair of dilators held in the right hand is inserted into the tracheal incision, which may now, if necessary, be enlarged by a touch of the knife as its edges are held on the stretch. If there is very much bleeding, the tube should be inserted at once, but otherwise it is preferable to turn the child on its side with the dilators in position until the hæmorrhage has ceased. The tube should be of Parker's pattern with a moveable shield, and it should be of such a calibre as to be grasped by the trachea and not roll about loosely inside it. This is an important point in regard to the safety of the after-treatment and the subsequent comfort of the patient, and it will be found that Nos. 24 and 26 are the most useful sizes; only in children under one year old have I commonly had occasion to use a No. 22. The smaller sizes are responsible for most of the difficulties that used to arise in the after-treatment of the case. The tube is fixed in position with tapes which are tied firmly round the neck, and a small pad of lint, spread with boric ointment on both sides, is placed underneath the shield, the mouth of the tube being covered loosely with a pad of gauze.

### Completing the Operation.

As far as the safety of the patient is concerned, the operation may be considered to have been practically completed as soon as the dilators have been inserted into the opening in the windpipe—that is to say, the urgent symptoms are then relieved and the operator can insert the tube quite at his leisure. This is really the only difficult part of the procedure, as it sometimes requires a little practice to get in a large tube. So long, however, as the tracheal dilators are not removed until the tip of the tube is felt to engage in the tracheal incision, no harm can come to the patient even from repeated attempts to insert a suitable tube.

The keynote to success in this method of performing tracheotomy lies in using the tactile sense of the left forefinger as a guide to the tracheal incision, rather than the eye; the advice given in more than one modern text-book of surgery that the trachea should not be opened until it can be seen and (what is more surprising) until all hæmorrhage has been arrested, is quite inapplicable to a tracheotomy for the relief of laryngeal obstruction in a child.

### Tracheotomy versus Intubation.

The chief advantages which tracheotomy performed in the manner which I have described possesses over intubation of the larynx are as follows:—

(*a*) It is infinitely easier.

(*b*) The relief is complete, inasmuch as almost the entire calibre of the trachea is restored to the patient. Intubation in a small child does not give sufficient room, and often results only in substituting one form of laryngeal obstruction for another.

(*c*) If there is any membrane loose below the larynx—and it is in any given case impossible to be sure that this condition is not present—it can be removed through the tracheotomy wound, but after intubation it remains and often blocks the tube completely.

(*d*) After tracheotomy the patient is much more comfortable than when intubation has been performed, and he can eat and drink without difficulty; after intubation the child has to be artificially fed in a special position by a skilled nurse.

(*e*) Should the canula become blocked with secretion, the nurse has only to take out, clean, and replace the inner tube, the outer sheath remaining *in situ* in the trachea; but when an intubation tube becomes obstructed it has to be withdrawn completely, and can only be replaced by the surgeon, who may not arrive in time.

(*f*) A tracheotomy tube can be left *in situ* as a rule until the obstruction to respiration has abated, but an intubation canula requires frequent changing if ulceration of the larynx is to be avoided.

In practice, tracheotomy is to be preferred to intubation, except when it is essential to avoid an external scar; or when it is practically certain that the laryngeal tube can be dispensed with in a few hours.

Coming now to the after-treatment, it is at first essential that the child should be allowed to sleep, and I have found, in practice, that nurses are apt to be too zealous in feeding the patient and in changing the inner tube. It is impossible to lay down any rule as to how frequently this latter procedure should be required in any given case, but I have known nurses who would change the tube whenever the child coughed. The chief guide is the character of the secretion from the trachea; if this is thin and watery, very little attention is required, but if it is scanty and tenacious, the inner tube may require to be changed very frequently; it is advisable to smear the canula with glycerine before replacing it in the latter type of case.

Then experience has shown that tracheotomy cases do very much better without a steam tent; if steam is used—and it is generally unnecessary—it should not be confined within a tent, but be allowed to issue from a jet which is placed near the cot. On no account should feathers be used while the tube is in place. They cannot be sterilised, and it has been found that broncho-pneumonia is very common when they have been pushed down the trachea with the intention of removing mucus which has accumulated below the tube. When a sufficiently large tube has been inserted, accumulation can be prevented by raising the foot of the cot on blocks.

In most cases the outer tube can be dispensed with on the third day, and the wound allowed to close gradually by itself. When it has been removed, a fair idea can be obtained as to whether it can be left out by closing the wound with a pad of lint and observing the character of the cough. If this indicates that the larynx is fairly patent, the tube need not be at once reinserted, but if the child does not use its larynx at all, any attempt to dispense with the tube should be avoided. If, also, there is any retraction of the edges of the wound with inspiration, it is advisable to replace the canula. When there is any difficulty in getting a nervous child to do without the tube, it is best to use a fenestrated canula with a hole at the top of the tracheal curve; the outer lumen of the tube can then be plugged and the child allowed to breathe through its larynx for periods which are gradually lengthened until laryngeal respiration is completely re-established.

Great care should be exercised in allowing a child to sit up after tracheotomy. In almost all cases at least three weeks in the recumbent position should be insisted on, and if the character of the pulse is at all rapid or intermittent, a longer period than this is advisable. Sudden heart failure in a diphtheritic patient is best treated by complete inversion of the child with simultaneous application of a hot sponge to the precordium. Valuable time is often lost by the administration of stimulants and hypodermic injections, which, as the circulation is temporarily in abeyance, do not reach their destination. This applies, of course, to all cases of diphtheria, and not only to those who have been tracheotomised.

Tracheotomy performed in the manner I have described is an operation that is suitable in emergency even when skilled assistance is not at hand. Provided that an anæsthetic is avoided, and that the operator's left forefinger is kept on the cricoid cartilage, the patient is safe, and the operator has no occasion for the perturbation which is—in my opinion not unjustly—associated with exposure of the trachea until it can be seen, with the coughing reflex abolished, and the right heart embarrassed by the administration of chloroform.

